# The interplay between plasticity and evolution in response to human-induced environmental change

**DOI:** 10.12688/f1000research.9731.1

**Published:** 2016-12-08

**Authors:** Sarah E. Diamond, Ryan A. Martin

**Affiliations:** 1 Department of Biology, Case Western Reserve University, Cleveland, OH, USA

**Keywords:** plasticity, evolution, human-induced, environmental change

## Abstract

Some populations will cope with human-induced environmental change, and others will undergo extirpation; understanding the mechanisms that underlie these responses is key to forecasting responses to environmental change. In cases where organisms cannot disperse to track suitable habitats, plastic and evolved responses to environmental change will determine whether populations persist or perish. However, the majority of studies consider plasticity and evolution in isolation when in fact plasticity can shape evolution and plasticity itself can evolve. In particular, whether cryptic genetic variation exposed by environmental novelty can facilitate adaptive evolution has been a source of controversy and debate in the literature and has received even less attention in the context of human-induced environmental change. However, given that many studies indicate organisms will be unable to keep pace with environmental change, we need to understand how often and the degree to which plasticity can facilitate adaptive evolutionary change under novel environmental conditions.

## Introduction and context

In the absence of sufficient compensatory mechanisms to cope with human-induced environmental changes, local extirpation of populations and possibly extinction of entire lineages will almost certainly occur ^1^. Quantifying organismal capacities for such compensatory mechanisms is therefore paramount to forecasting responses to ongoing and future human-induced changes to the environment ^2^. Of particular importance is quantifying the relative contributions of phenotypic plasticity, where a single genotype produces a range of different phenotypes in response to environmental variation, and evolution, where additive genetic differences underlie phenotypic change across generations or among populations. This distinction is critical, as plasticity and evolution often occur over different timescales and can operate under different constraints ^3^.

Certainly much progress has been made over the last few decades in disentangling plastic from evolved changes in phenotypes under human-induced environmental change ^3– 12^. Yet the distinction of plastic versus evolutionary contributions in a given population at a given time may be an oversimplification, as these mechanisms can interact to shape phenotypic changes in populations over time ^6^. Basic research on evolution has long debated whether plasticity facilitates or constrains adaptive evolution ^13^. However, this idea has only recently come into focus for theory ^14, 15^ and empirical work ^16, 17^ involving responses to human-induced environmental change. Here, we briefly review the interplay between plastic and evolutionary responses to human-induced environmental change and highlight new research areas for future development on the role of plasticity in shaping evolutionary responses to changes in climate, land-use, and environmental toxins. While we consider these environmental stressors separately for organizational purposes in this review, we acknowledge that many of these factors are not mutually exclusive, e.g. land-use changes can often include changes in environmental toxicity.

## Phenotypic plasticity as a facilitator versus constraint on evolution

Evolutionary theory predicts that natural selection on environmentally insensitive (canalized) traits imposed by novel environments will drive populations toward their new local fitness optima ^4^, yet evolution in this sense might not achieve large enough shifts in trait values or be fast enough to keep pace with the scale and rapidity of human-induced changes to the environment before populations undergo extirpation. Limits on the amount of standing genetic variation in populations, mutation rates, fitness tradeoffs, the strength and consistency of selection, and the genetic correlations among traits can all serve to slow the rate of evolutionary change ^5^.

Likewise, there are limits on shifts in trait values owing to existing phenotypic plasticity. Most traits exhibit phenotypic plasticity. However, plasticity may simply fail to produce shifts in phenotypes of a great enough magnitude to cope with rapid environmental change ^18^. There is also no guarantee that plasticity will be adaptive for the novel conditions generated by human-induced environmental change ^19^. Whether plasticity is adaptive or not depends on whether the environmentally induced phenotypes are closer or farther from the local optimum. Because novel environments are, by definition, environments that organisms have never experienced previously, it is entirely plausible that adaptive plasticity in current environments will be maladaptive in the novel environment ^20, 21^. This prediction should, however, be tempered to some degree, as novelty can arise not only through environments not previously experienced but also through alterations to the frequency of environmental conditions experienced (e.g. 22); in the latter case, organisms may be more likely to evolve adaptive plasticity, as they are responding to frequency differences rather than absolute differences in their environment.

**Figure 1.**
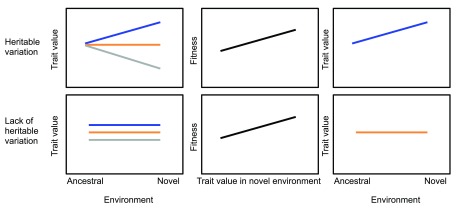
Adaptive trait evolution in a novel environment. When a population encounters a novel environment (for example, novel urban and ancestral rural habitats), it can either express hidden, habitat-dependent heritable variation in a given trait (top row) or not (bottom row). In the case where this variation is expressed, selection (middle column panels) can refine this phenotypically expressed genetic variation into an optimal canalized trait. In the absence of habitat-dependent genetic variation (and assuming a lack of novel mutation), the population cannot evolve towards the new trait optimum.

Given that existing plasticity and evolution may on their own be insufficient to cope with environmental change, there has been renewed interest in the case where novel environments reveal cryptic genetic variation on which selection can act to refine the form and regulation of this novel phenotypic variation ( Figure 1) ^23^. Plasticity may be a key source of evolutionary innovation, allowing organisms to cope with the large magnitude and rapid rate of human-induced environmental changes (the plasticity-first mechanism of evolutionary change; see 13). Indeed, plasticity may be an especially efficient source of variation, as the effects of environmental novelty can reach each individual of the population, unlike mutations, which arise in a single individual and are then likely to disappear ^24^. Despite the potential importance of the plasticity-first mechanism in the context of human-induced environmental change, there are relatively few empirical data that address its components, coupled with abundant controversy in the literature over plasticity’s role in shaping evolutionary responses to environmental novelty.

Evidence is accumulating in support of the hypothesis that novel environments reveal cryptic genetic variation on which selection can act ^25^. Recent meta-analyses suggest that novel environments considered broadly tend to reveal cryptic genetic variation ^26^, as do those that focus specifically on human-induced novel environments ^27^. However, most of this human-induced environmental novelty involves variation in resource quality and quantity, representing a small subset of the environmental novelty induced by climate change, land-use change, and environmental toxins that are the focus of this review. As a consequence, while there is evidence that human-induced environmental change can reveal cryptic genetic variation, a broader range of environmental changes needs to be examined to establish the generality of this pattern. Nevertheless, laboratory selection experiments have shown that this uncovered plastic variation can facilitate evolutionary change via genetic accommodation ( Box 1), which describes the evolution of environmentally induced phenotypes, including the evolution of canalized traits from initially plastic traits through the process of genetic assimilation ^23, 28, 29^. Despite this, considerably fewer studies have tested the relationship between initial plasticity and subsequent evolution in natural populations (but see for example 30, 31).

Box 1. **Plasticity’s role in shaping evolution.**

How plasticity can constrain evolution:

- If plasticity in novel environments results in high mean fitness (i.e. adaptive plasticity), then plasticity can weaken subsequent selection in the novel environment and hide genotypic variation from selection ^86^. The occurrence of adaptive plasticity in novel environments is likely to occur only when the novel environment is similar to native or ancestral environments and therefore a product of past selection ^87^.
- Plasticity may drive a population to extirpation in novel environments before adaptive evolution can occur if plasticity results in very low mean fitness (i.e. maladaptive plasticity), for example through developmental instability or a breakdown in homeostasis ^86, 87^.

How plasticity can facilitate evolution:

- Plasticity may facilitate evolution in novel environments when it acts to buffer populations from extirpation long enough for selection to act on standing or cryptic genetic variation ^86, 87^.
- Gene by environment interactions may reveal **cryptic genetic variation** in novel environments when environmental variation falls outside the range generally experienced, exposing heritable phenotypes that were not expressed in the ancestral environment to selection. Since the speed of evolution is in part dependent on the degree of genetic variation underlying phenotypic traits under selection, evolution may proceed more rapidly when novel plasticity is revealed ^24, 88, 89^.

How plasticity itself can evolve:

- Plasticity can evolve when selection acts on genetic variation in the phenotypic expression of individual genotypes across environments (i.e. reaction norms). This process has been named **genetic accommodation** ^24^. Under this broad umbrella, selection may act on genetic variation to change the slope of the reaction norm or the mean of a trait expressed across environments ^90^. Selection to canalize the expression of a novel environmentally induced phenotype has been specifically termed **genetic assimilation** ^28, 91, 92^, and its reverse, the restoration of the ancestral phenotype in a novel environment, has been termed **genetic compensation** ^20^.

Human-induced novel environments are excellent sources to look for the exposure of cryptic genetic variation and to explore plasticity’s role in shaping evolution, but comparatively few studies have taken advantage of these opportunities ^31^. On the one hand, human-induced changes to the environment yield an imperative to understand plastic and evolutionary responses to new environments for the goal of biodiversity forecasting and conservation planning, but on the other hand, these often-rapid human-induced environmental changes may help overcome historical challenges in assessing plasticity’s role in evolutionary change. Specifically, many of the comparisons between ancestral and novel populations represent divergence processes that are relatively old ^32^. Rapid human-induced environmental changes provide access to the early stages of plastic and evolved responses to new environments. Human-induced environmental novelty may also be more straightforward for attribution—that is, determining the proximate drivers of changes in the environment—as compared with historical comparisons between ancestral and novel populations where attribution is perhaps less clear and there is more time for suites of different environmental changes to accumulate.

In this review, we discuss recent work on the interplay between plastic and evolved responses to human-induced environmental change. Specifically, we consider whether plasticity creates novel opportunities for selection to act, thereby facilitating evolution, or whether plasticity buffers environmental variation, dampening selection and constraining evolution ( Box 1). Because we are covering a diverse array of changes from global climate change to environmental toxins with the aim of stimulating discussion on plastic and evolutionary responses to these stressors, we take a case study approach rather than an exhaustive review, highlighting recent and transformative work in the field.

## Existing plasticity, evolutionary potential, and the role of plasticity in shaping evolutionary responses to human-induced environmental change

### Climate change

Humans have made lasting impacts on the environment for thousands of years, from megafaunal extinctions during the late Quaternary period ^33^ to rapid industrialization and global temperature rise within the last century ^34^. The magnitude of recent human-induced changes, particularly to climate, has already led to the global redistribution of plants and animals in space and time ^35, 36^. For those organisms that cannot get out of the way of climate change, they must rely instead on plastic and evolved responses. A major focus has been on whether current amounts of phenotypic plasticity and evolutionary potential are sufficient to keep pace with projected climate velocities ^37, 38^. Of course, there are many effects of contemporary global climate change—from atmospheric deposition of elemental compounds to shifts in precipitation patterns—but temperature is perhaps the best studied and one that impacts organisms from biochemical rate processes up through species interactions, community dynamics, and ecosystem function ^39^.

By the end of the century, global temperature is expected to rise by over 4°C under the highest greenhouse gas emissions scenarios, which we are currently tracking ^34^. Studies of organismal capacities to cope with this change, either through plasticity or evolution, yield mixed evidence for whether organismal changes will be large enough and proceed fast enough to keep pace with climate change ^18, 38, 40, 41^. In general, there has been a strong research emphasis on existing plasticity in phenology, morphology, physiology, and behavior, perhaps because of the assumption that plasticity will operate over a timetable commensurate with rapid global climate change and perhaps also stemming from the added challenges of assessing evolutionary responses ^42^. This emphasis is not without merit, as plasticity has been broadly shown to be a critical component of the response to climate change ^43^. However, existing plasticity is not always sufficient to cope with climate change. For example, Anderson and colleagues found a substantial role for phenological plasticity in plant flowering time under recent climate change but concluded that plasticity alone is insufficient and evolutionary change is needed for these populations to keep pace ^44^. While such variance partitioning studies of responses to climate change among plastic and evolved components is growing, there remain comparatively few studies assessing how this plasticity shapes evolutionary responses ^3^.

Those studies that have explored the role of plasticity in facilitating adaptive evolutionary responses to climate change have found little evidence in support of this mechanism, though this may be as much a product of the organisms and systems for which these data are available as it is a general biological pattern. For example, research on lizard responses to climate change from Buckley and colleagues indicates that plasticity in thermoregulatory behavior constrains lizard evolutionary responses to warming by shielding variation in thermal performance from selection ^45^. As a notable contrast, Logan and colleagues found evidence of selection on thermal performance traits when lizards were transplanted to a novel thermal environment ^46^, but plasticity’s role in facilitating or constraining evolutionary change was not reported. It is worthwhile to consider why there are so few studies on genetic accommodation under climate change. One important limitation may be how the ancestral and novel populations are assessed under a temporal change in climate. Resurrection studies or studies which compare the leading edge of a rapid range expansion to the center range seem to be strong candidates for such an analysis ^47^. Next, we consider two environmental changes—land-use and pollution—where the ancestral and novel populations are readily accessible.

### Land-use change

Although there are many types of land-use changes occurring, including deforestation and habitat fragmentation, to which organisms respond through plasticity and evolutionary change ^48^, urbanization is an increasingly important source of land-use change. Rates of urbanization are accelerating globally, with current levels of urbanization at three percent of the Earth’s landmass, excluding Greenland and Antarctica ^49^. This may not seem like an exceptionally large number, but over half of the world’s population lives within these urbanized areas, and pockets of urbanization dot almost every corner of the globe ^50^. Although studies have begun to quantify community composition and phenotypic changes in populations across urban and nearby rural habitats, few have explored the mechanisms that contribute to these changes. Because the urbanization process offers both novel urban habitats in close spatiotemporal proximity to ancestral rural habitats and the potential for replication across independent ancestral-novel comparisons ^51, 52^, urbanization gradients are excellent, but underused, sources of ancestral-novel comparisons needed for assessing plasticity’s role in shaping evolutionary change. Of particular interest is the rapid change in environmental temperature over space and time under the urbanization process, especially as urban warming can serve as a space-for-time proxy to understand responses to global climate change. Urban heat island effects are relatively consistent across many regions, with temperature increases in excess of several °C in the air column and over 10°C at the surface being possible (though there are exceptions, especially urbanization in desert habitats ^53^). Though, of course, many other changes accompany the urbanization process, including changes in habitat structure, nutrient availability, and pollution ^54^.

In general, studies that explore plastic and evolutionary responses to urbanization have lagged behind climate change studies. This is surprising, since one of the best-known and earliest examples of rapid evolutionary change, industrial melanism in peppered moths, was itself driven by the fast progression of urbanization (and environmental pollution) associated with the industrial revolution ^55, 56^. More recently, Donihue and Lambert ^54^ provided an excellent overview of how to assess and disentangle plastic and evolved responses to urban land-use change in addition to an exhaustive review of the studies that have done so in urban environments. They defined three criteria for demonstrating adaptive evolutionary change in response to urbanization, including quantifying phenotypic shifts in traits, measuring the fitness effects and genetic basis of those traits, and identifying drivers of trait changes. Only two studies met these criteria at the time of the review, including one study on killifish responses to elevated polychlorinated biphenyl pollution in urban environments ^57^ and another on the reduction of dispersing versus non-dispersing plant seeds in urban environments ^58^. Notably, the killifish study highlights the fact that the environmental stressors we consider in our review—climate change, land-use change, and environmental toxins—are not mutually exclusive, as, for example, killifish responses to polychlorinated biphenyl compounds may be considered in context of both land-use change and environmental toxicity. Relatedly, structural changes in urban environments, specifically replacing grassland and forest with impervious surfaces like roads, sidewalks, and buildings, contributes to the urban heat island effect; as the study on plant seed production demonstrated, these structural changes can also serve as an agent of selection themselves. Dispersing seed pods are selected against in urban environments because there are large gaps in suitable habitat owing to intervening sidewalks and roads ^58^.

Evidence consistent with an interpretation of urban evolution continues to accumulate. For example, Winchell and colleagues ^59^ have found phenotypic shifts in lizard morphology in urban areas where lizards use artificial surfaces as perches as opposed to vegetation in unaltered habitats (an interesting parallel with the structural changes in urban habitats altering plant seed dispersal); a common garden experiment suggested a genetic basis for these changes in morphology, though the adaptive nature of these changes has yet to be demonstrated, and maternal effects cannot be fully excluded with their use of first-generation offspring from field-collected parents. Interestingly, evolutionary responses to urban heat islands appear to be one of the least-studied aspects of the urbanization process despite the ubiquity of the urban heat island signal among cities. Angilletta and colleagues ^60^ demonstrated an increase in upper thermal tolerance in city ants compared with rural ants, though it is unclear whether this shift is adaptive and has a genetic basis. McLean and colleagues used a common garden experiment to demonstrate a genetic basis for shifts in growth rate of a chitinolytic fungus along an urbanization gradient, though, again, the adaptive nature of these changes is unclear, and only one urban-rural comparison was made ^61^. Although urban evolution is gaining traction in the literature, the use of urban environments to assess the role of plasticity in shaping evolutionary responses has been used only to a marginal extent and remains open for future development. In particular, replicated urban-rural comparisons with multigenerational common garden studies are needed to explore the potential for urban evolution and its interplay with plasticity.

### Environmental toxins

In conjunction with increasing CO _2_ emissions and changes in land-use, more than a century of industrialization has led to the accumulation of toxins in the environment, notably heavy metal and pesticide contaminants ^62^. Environmental toxins can have severe negative consequences for organismal fitness and population survival through a diverse range of effects and mechanisms of action ^63^. Despite this diversity and in contrast to urbanization and climate change, the evolution of resistance to environmental toxins is well established ^64– 66^: for instance, the classic example of divergent evolution and speciation of plant populations living in and adjacent to toxic mine tailings ^67– 70^.

Phenotypic plasticity also contributes to organismal responses to environmental toxins, as prior exposure to heavy metals ^71^ and pesticides ^72^ can reduce the negative effects of later exposure. Interestingly, exposure to one toxin may induce a plastic response with positive fitness effects (i.e. an adaptation) to other toxins ^73, 74^, but the generality of this inducible “cross-tolerance” is currently unknown. This shared adaptive plasticity does not appear to be restricted to toxins with common mechanisms of action, contrary to expectations ^73^. Indeed, this result suggests an important role of plasticity in the evolution of toxin tolerance, as cross-tolerance would induce initially adaptive rather than maladaptive plasticity in response to novel toxins, possibly facilitating subsequent evolutionary refinement.

Like urbanization, the spatiotemporal heterogeneity of environmental toxins provides an opportunity to evaluate the role of plasticity in shaping evolution. Indeed, comparisons among populations differing in their exposure to pesticides provide some of our best evidence that genetic assimilation plays a role in evolutionary responses to anthropogenic change. Historically, studies focused on the evolution of tolerance in pest species directly targeted by pesticide application. A recent emphasis on non-pest species incidentally affected by pesticides has provided novel insights into the mechanisms underlying pesticide tolerance. By comparing pesticide tolerance among naïve populations to populations exposed to pesticides in contaminated environments (e.g. populations close to agriculture), numerous studies have now found evidence for the evolution of greater resistance to pesticides in affected populations of anurans and aquatic invertebrates compared to naïve populations ^73, 75– 83^.

Using an agricultural land-use gradient in a space-for-time substitution, Hua and colleagues recently extended these findings to test key predictions of genetic assimilation ( Box 1): the canalization of inducible phenotypic variation by the action of natural selection. By quantifying both evolved and inducible (i.e *.* plastic) tolerances of wood frog populations varying in their distance from agriculture to an insecticide, Hua and colleagues found that evolved tolerance to a potentially lethal exposure of insecticide decreased with distance from agriculture ^76, 79^. In contrast, the degree of plastic tolerance induced from an initial sublethal exposure increased with distance from agriculture ^76^. Together, these results are consistent with the predictions of genetic assimilation; exposure to novel environments induced the expression of phenotypic variation in ancestral populations, and this variation was then canalized in populations with a history of exposure to the novel environment ^28, 29^. We emphasize that while these studies provide indirect evidence of genetic assimilation, more direct tests are needed. To accomplish this goal, resurrection studies and the study of extant populations varying in their time of exposure to novel environments may provide powerful means of testing the role of plasticity and evolution for responses to human-induced environmental change.

## Ways forward: improving our understanding of plastic and evolutionary responses to a rapidly changing world

That plasticity is a powerful mechanism for allowing organisms to cope with rapid human-induced environmental change has widespread empirical support. Evidence in support of rapid evolution in response to environmental change is less abundant ^3^ but accumulating. Although quantifying organismal capacities to respond to environmental change through plasticity and evolution is and should continue to be a major research priority, a key shortcoming in this area is a limited quantity of empirical data on the interaction of these mechanisms, specifically the role of plasticity in shaping evolutionary responses to environmental change ^31^. Climate change, land-use change, and environmental toxins provide conservation and biodiversity forecasting challenges; however, these agents of environmental change also provide opportunities to explore the potential for novel environments to release cryptic genetic variation and how selection might act on that variation to enact evolutionary change. To move forward, we suggest researchers should especially focus on (i) quantifying the expression of cryptic genetic variation in the wild, (ii) assessing patterns of selection on cryptic genetic variation in natural populations, and (iii) quantifying the strength and direction of evolutionary versus plastic responses to novel environments. An especially powerful approach may be assessing evolutionary and plastic responses across a gradient of time rather than space. By identifying and studying populations exposed to novel selective environments for different lengths of time using multi-generational common garden designs, we can directly assess the mechanisms underlying these responses. Human-mediated changes via urbanization and environmental toxins may be especially amenable to such an approach where historical records can be harnessed to calibrate temporal variation in the selective environment.

In addition to the issues with plastic and evolutionary responses to environmental change that we considered here, several related areas are also seeing further developments. One of these areas concerns the covariation between selection and genetic variation when both are affected by the same environmental factor ^27, 84, 85^. A major assumption underlying the genetic accommodation mechanism is that there will be a positive relationship between the degree of genetic variation revealed and the strength of natural selection in novel environments. However, this outcome is not guaranteed, and it is possible that genetic variance could be lower for the targets of selection when selection is stronger; in practical terms, this means that evolution could be slower, as trait heritability (the ratio of additive genetic variance to total phenotypic variance) would be lower in the novel environment despite stronger selection. There have been very few empirical examinations of the nature of the covariation between the amount of variation exposed and the strength of selection in novel environments. Wood and Brodie have examined this relationship with several agents of selection, including variation in environmental temperature ^27^. Interestingly, while the authors found the strength of selection to vary with temperature, this variation was not correlated with genetic variance, as greater and lesser values of genetic variance were detected with increasing strength of selection. In contrast, environmental novelty, as defined by the study authors, consistently revealed increased genetic variance but the studies synthesized by Wood and Brodie did not concurrently measure selection in these novel environments. Going forward, we need additional empirical data on both the strength and targets of selection and the degree of heritable variation available for selection to act to determine when and how the assumption of a positive correlation between strength of selection and revealed genetic variance in novel or changing environments is upheld or broken. Evolution on contemporary timescales in response to human-induced environmental change provides at least as many opportunities to explore fundamental questions on interactions among plasticity, organismal ecologies, and the magnitude and direction of evolutionary change as it does challenges in predicting complex responses to environmental change for conservation planning.
